# Determination of Proteinaceous Selenocysteine in Selenized Yeast

**DOI:** 10.3390/ijms19020543

**Published:** 2018-02-11

**Authors:** Katarzyna Bierla, Ryszard Lobinski, Joanna Szpunar

**Affiliations:** Institute of Analytical Sciences, IPREM, UMR 5254, CNRS-UPPA, Hélioparc, 2 Avenue Angot, 64053 Pau, France; katarzyna.bierla@univ-pau.fr (K.B.); ryszard.lobinski@univ-pau.fr (R.L.)

**Keywords:** selenized yeast, selenocysteine, HPLC-ICP MS, HPLC-ESI MS

## Abstract

A method for the quantitation of proteinaceous selenocysteine (SeCys) in Se-rich yeast was developed. The method is based on the reduction of the Se-Se and S-Se bridges with dithiotretiol, derivatization with iodoacetamide (carbamidomethylation), followed by HPLC-ICP MS. The chromatographic conditions were optimized for the total recovery of the proteinaceous selenocysteine, the minimum number of peaks in the chromatogram (reduction of derivatization products of other Se-species present) and the baseline separation. A typical chromatogram of a proteolytic digest of selenized yeast protein consisted of up to five peaks (including SeMet, carbamidomethylated (CAM)-SeCys, and Se(CAM)_2_) identified by retention time matching with available standards and electrospray MS. Inorganic selenium non-specifically attached to proteins and selenomethionine could be quantified (in the form of Se(CAM)_2_) along with SeCys. Selenocysteine, selenomethionine, inorganic selenium, and the water soluble-metabolite fraction accounted for the totality of selenium species in Se-rich yeast.

## 1. Introduction

Selenium is an essential micronutrient which plays a vital role in many of biochemical and physiological processes, including immune function, thyroid hormone metabolism, and antioxidant defense systems [1]. The deficiency of selenium is a world-wide problem which is addressed by supplementation [2]. Selenium-enriched yeast is a popular food supplement and an established ingredient of Se-enriched premixes and finished feed products [3,4,5,6]. The understanding and optimization of the biochemical processes of Se incorporation during yeast growth and the characterization of the final products requested by regulatory agencies require the development of suitable methods for speciation analysis [7,8].

Selenomethionine (SeMet) concentration is an established parameter of the “organic” character of selenium-rich yeast food and feed supplements [7]. Its determination can be validated by the use of SELM-1 standard reference material (National Research Council of Canada) although the physicochemical properties of yeast produced by different methods may vary from the SELM yeast [7,9]. Selenocysteine was long believed to be absent in the protein fraction because of the absence of the UGA codon in *Saccharomyces cerevisiae* and was quantified uniquely in the water-soluble metabolite fraction as the sum of Se-glutathione tripeptides (which were at the time the only SeCys-containing species identified) [10]. A recent study demonstrated, however, that, in spite of absence of UGA codon, selenocysteine (SeCys) can be found in Se-rich yeast proteins [8]. Its origin would be a non-specific replacement of sulphur to selenium on the pathway of cysteine synthesis [11,12]. The Se/S substitution ratio in cysteine, estimated as an average for 26 peptides issued from the tryptic digestion of 19 Se-containing proteins located in a 2D electrophoretic gel by laser ablation-ICP MS imaging, was 14.1 ± 4.8% [7]. This result was not validated by the direct SeCys determination because of the lack of a reliable analytical method.

Indeed, a critical analysis of the literature reports on the SeCys determination in Se-rich yeast showed several problems related to the identification of the SeCys peaks and its quantification [10]. SeCys was often confused with its dimer (selenocystine, SeCys_2_) which was used to quantify selenocysteine by standard addition. Consequently, SeCys_2_ was reported several times not only after proteolytic digestion, but also after simple aqueous extraction [13,14,15,16,17] which would assume either the free availability of this dimer, or an almost complete proteolytic autolysis. Moreover, the literature contains no indication that the proteolytic digestion may lead to the liberation of the SeCys_2_ dimer from peptides or proteins, which is the prerequisite of producing a species to be matched with the SeCys_2_ standard. HSeCys-containing peptides are known to be unstable leading to the loss of Se and production of dehydroalanine [18]. The lack to address properly these questions did not prevent a considerable number of data to be reported for yeast samples which vary from 0.5% [19] to 35% [20].

Many questions related to the SeCys determination were addressed for in animal tissues, eggs, and human serum which contain predominantly genetically-encoded SeCys [21,22,23,24]. SeCys-residues in proteins are highly reactive and readily form Se–Se and Se–S bonds with other SeCys– and Cys residues, respectively. Reduction of these bonds with dithiotretiol and capping the SeCys residues by carbamidomethylation to prevent them from reacting again, followed by complete proteolysis yielded carbamidomethylated SeCys (CAM–SeCys), which could be quantified by HPLC-ICP MS. The method was validated using a control sample enriched with a selenoprotein (glutathione peroxidase) standard and the quantitative mass balance of all the selenium-species recovered.

The extrapolation of this method to Se-rich yeast is not straightforward for several reasons. First, Se-rich yeast contains a considerable metabolite fraction accounting for up to 20% of the total selenium and containing over 100 metabolites [25,26]. Several of these metabolites are likely to react with iodoacetamide upon reduction producing complex chromatograms at, and in, the vicinity of the SeCys signal making the baseline resolution and quantification of SeCys impossible. Second, the 100–1000 times higher SeCys expected concentration in Se-rich yeast prevents the use of the expensive glutathione peroxidase standard for validation and, additionally, the different speciation of non-specifically inserted SeCys in comparison with SeCys in true selenoproteins would make such a validation questionable.

The goal of this research was to develop an analytical method for the quantitative determination of proteinaceous selenocysteine in Se-rich yeast. The protein fraction was obtained by the removal of low-molecular mass (<1000 Da) metabolites. The S–S, Se–Se, and Se–S bridges were reduced with dithiothreitol, the free thiols and selenols were capped by carbamidomethylation and the proteins were subjected to proteolysis to produce amino acids. The Se speciation in the post-reaction mixture was probed by HPLC-ICP MS to account for the mass balance of Se-species in the water-insoluble fraction containing proteinaceous SeCys, SeMet, and inorganic selenium. Electrospray MS/MS served to identify the unknown species. The CAM–SeCys peak was used for quantification.

## 2. Results and Discussion

### 2.1. Characterisation of the Samples in Terms of Total Selenium, Total Selenomethionine, and Total Selenometabolite Fraction

The currently available methodology for the Se-rich yeast analysis allows the determination of the total selenium (by sample digestion and ICP MS), total selenomethionine (by proteolysis and anion-exchange HPLC-ICP MS), and the total water-soluble selenium referred to as “selenometabolite fraction” [7]. These data for the samples studied in this work are given in Table 1. The “protein fraction” of the samples was 81–87% and the SeMet concentration 60–85%. Part of selenium remains, therefore, unaccounted for and was attributed to selenocysteine on the basis of the proteomics data [8]. It was attempted here to develop a method allowing its quantification.

### 2.2. Concerns About Selenocysteine Determination

#### 2.2.1. Recovery

Whereas SeMet can be recovered quantitatively by proteolysis in the presence of a reducing reagent (2-mercaptoethanol or dithiothreitol), selenocysteine in this conditions is largely lost by the formation of dehydroalanine [27]. Another risk is the risk of oxidation producing different species such as, e.g., SeCys–Cys and SeCys–SeCys dimers, which elute in the void of reversed phase HPLC. Additionally, the chromatographic recovery of the reduced SeCys is not quantitative because of its reactivity with the column stationary phase. Therefore, the quantitative recovery of selenocysteine is critically dependent on the capping of highly-reactive selenol (–SeH) groups which can be achieved by carbamidomethylation [21,22,24].

#### 2.2.2. Chromatographic Baseline Resolution

The highest resolution for Se-compounds present in yeast is achieved by reversed-phase HPLC using ion-paring reagents in the mobile phase [28]. These conditions are responsible for the considerable electrospray ionization suppression in addition to the generally poor ionization of amino acids. Therefore, detection by ICP MS is privileged. It is selenium-specific, but requires baseline resolution of carbamidomethylated SeCys from other selenium compounds and products of their reaction with iodoacetamide. The ICP MS detection has the advantage of the similar response of the different selenium species which allows their quantification with a single calibration standard (in the range of the isocratic elution) and facilitates the determination of the Se mass balance.

**Figure 1:**
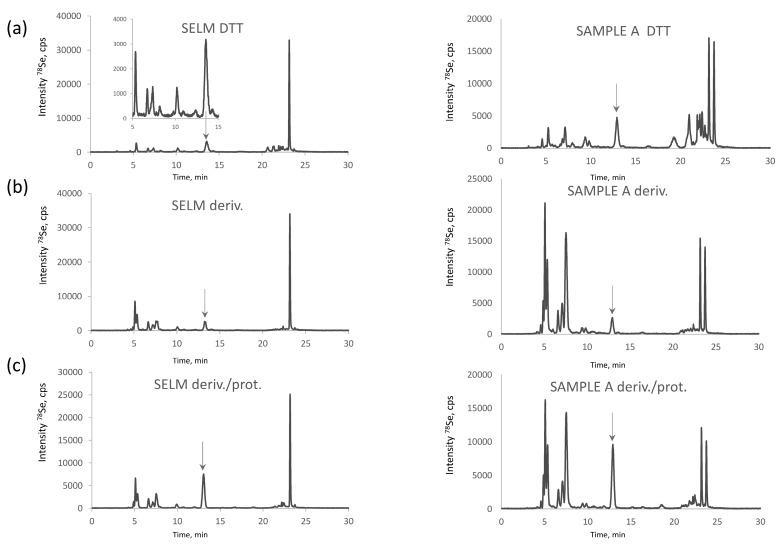
Chromatograms of selenometabolite (water-soluble) selenized yeast fraction (**a**) raw (upon addition of 30 µL of DTT solution); (**b**) derivatized with IAM; and (**c**) derivatized and digested with protease obtained for SELM-1 (left panel) and a commercial selenized yeast sample (right panel).

**Figure 2:**
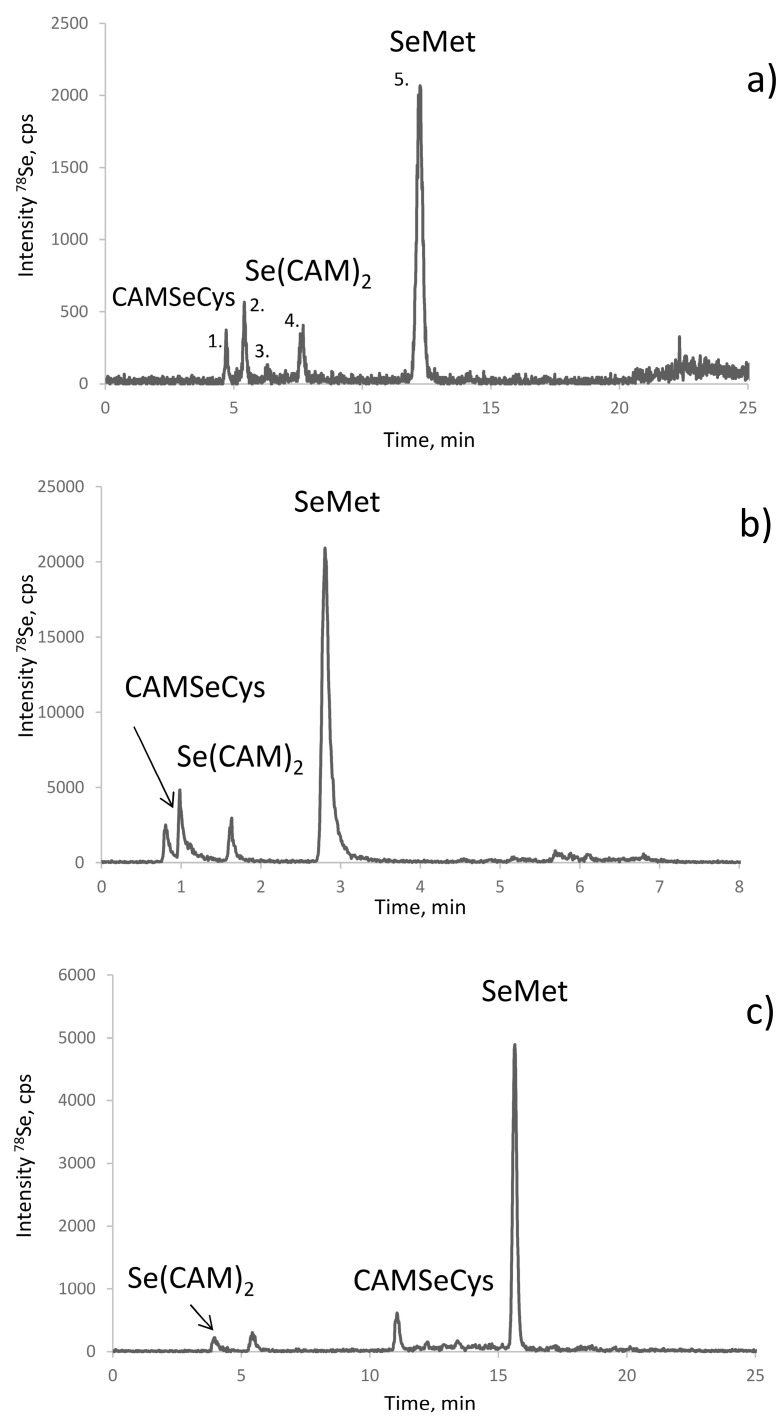
Chromatograms obtained for SELM-1 in optimized conditions (detailed in Table 2): (**a**) RP (C8) HPLC-ICP MS; (**b**) CS C18 AQ HPLC-ICP MS; and (**c**) Hypercarb HPLC-ICP MS.

Se-rich yeast is an extremely complex sample in terms of Se-speciation. More than 100 Se compounds were reported in the metabolite fraction, usually accounting for 10–20% of total selenium in *Saccharomyces cerevisiae* [7,26]. Figure 1a shows HPLC-ICPMS chromatograms for the selenometabolite fraction for the SELM-1 reference material and one of the samples studied. Most of the species (Figure 1b) react with iodoacetamide producing a crowd of peaks in the retention time area of CAM-SeCys (cf. Figure 2), rendering baseline separation of CAM-SeCys virtually impossible, or at least unreliable.

#### 2.2.3. Distribution of SeCys and SeMet between the Metabolite and Protein Fraction

The proteolysis of the metabolite fraction indicates the quasi-absence of proteinaceous selenoamino acids. The signal of selenomethionine (Figure 1c) corresponds to about 6% of selenium recovered which corresponds to ca. 1% of the total selenium present in the sample. This observation is in agreement with the earlier studies of the characterization of the water soluble protein fraction of yeast which accounted for 3–5% of the total metabolite fraction, i.e., 0.3–1% of the total selenium present and was distributed among the SIP18 and HSP12 proteins [29]. Consequently, the presence of proteinaceous selenoamino acids in this fraction can be neglected and this fraction can be discarded prior to the SeCys determination.

### 2.3. Optimization of the Baseline Separation of CAM-SeCys in the Proteolytic Extract

The objective of optimisation was to achieve the elution of CAM-SeCys away from the void of the column and its baseline separation from other Se-containing molecules. The use of an ion-pairing reagent, such as heptafluorobutyric acid (HFBA) [30,31], was essential to improve the retention and the separation. Figure 2 shows chromatograms obtained using different columns in the optimized conditions. The most defined separation was obtained using the AltimaC8 column which shows five well resolved Gaussian-shape peaks eluting isocratically at 3% of methanol (Figure 2a). Note that these peaks remain separated using the same stationary phase in the UPLC format (Figure 2b) while the elution time is reduced five-fold. The use of the HyperCarb (porous graphitic carbon) is an interesting alternative for the validation of the separation as CAM-SeCys eluted far away from the void and artefact compounds (Figure 2c). The elution of selenium was quantitative (90–102% when compared to the total selenium content in the sample).

### 2.4. Identification of the Peaks Observed

**Figure 3:**
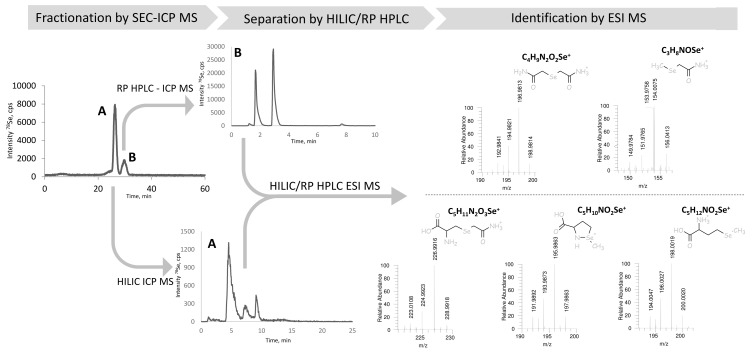
The scheme of the chromatographic purification selenium species in the proteolytic digest prior to HPLC-ESI MS identification including: initial fractionation of the proteolytic digest by SEC-ICP MS, separation of the resulting fractions by HILIC (fraction **A**), and RP (C18) HPLC (fraction **B**) with dual ICP MS/ESI MS detection, leading to the identification of selenium species.

The identification of all the chromatographic peaks observed was considered to be essential to understand the reactions taking place and validate the method. The use of electrospray MS/MS in parallel and matching the chromatograms with ICP MS and ESI MS/MS detection, as proposed elsewhere [32,33], failed because of the presence of the ion-pairing reagent (HFBA) and the large quantity of iodine due to the excess of iodoacetamide. Therefore, the post-proteolysis mixture was fractionated by size-exclusion chromatography (SEC) to remove the excess of iodine using the chromatographic conditions detailed in Table 2. The outline of these experiments is presented in Figure 3. The Se-containing fractions were collected between 22.3–25.17 min (fraction A) and 28–31.7 min (fraction B), lyophilized, and re-dissolved in water. Aliquots of fraction A and B were injected into a C8 column using ICP MS detection (chromatograms not shown). Fraction A contained peaks 1, 3, and 5, and fraction B peaks 2 and 4, referred to by these numbers in Figure 2a. Several chromatographic conditions were then attempted to obtain the separation of the compounds for their dual detection by ICP MS and ESI MS/MS.

For fraction A (peaks 1, 3, and 5 in Figure 2a) a positive result was obtained by hydrophilic ion interaction chromatography (HILIC). The three peaks identified correspond to CAM-SeCys (*m*/*z* 226.9916), cyclized SeMet species (*m*/*z* 195.9864) characteristic for terminal SeMet [34] and SeMet (*m*/*z* 198.0019). This identifies unambiguously peaks 1 and 5 as originated from SeCys and SeMet, respectively, and the minor peak 3 to be a SeMet ring form. For Fraction B (peaks 2 and 4) two compounds could be separated using a reversed-phase C18 (Zorbax) column. The molecular masses confirm the presence of derivatized inorganic selenium: dicarbamido-methylselenium (C_4_H_9_N_2_O_2_Se^±^, *m*/*z* 196.9813) (peak 2 in Figure 2a) and carbamidomethyl-selenium (*m*/*z* 153.9758) being a SeMet fragment and derivatization reaction artefact.

**Figure 4:**
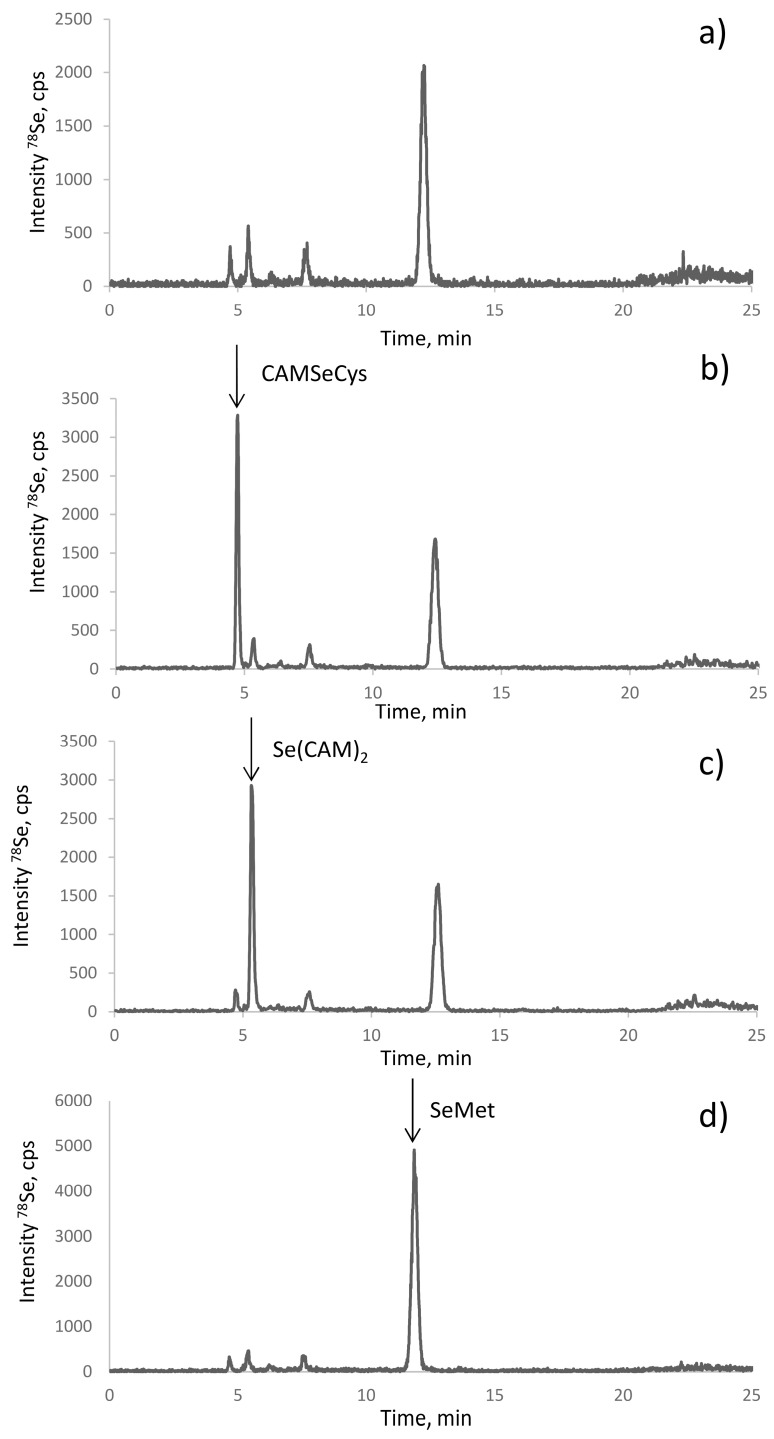
Identification of the species in the proteolytic digest of selenized yeast (SELM-1) (**a**) using spiking with standards of (**b**) CAM-SeCys, (**c**) Se(CAM)_2_, and (**d**) SeMet.

The electrospray MS results were validated by the spiking experiments. The retention times of the standards of CAM-SeCys, Se(CAM)_2,_ and selenomethionine matched with the putative peak assignments (Figure 4). The standard additions also allowed the quantification of the respective compounds. Their response was identical, which confirmed the possibility of using a single standard (SeMet) for quantification of all the species.

### 2.5. The Origin of Dicarbamidomethylselenium (Se(CAM)_2_)

**Figure 5:**
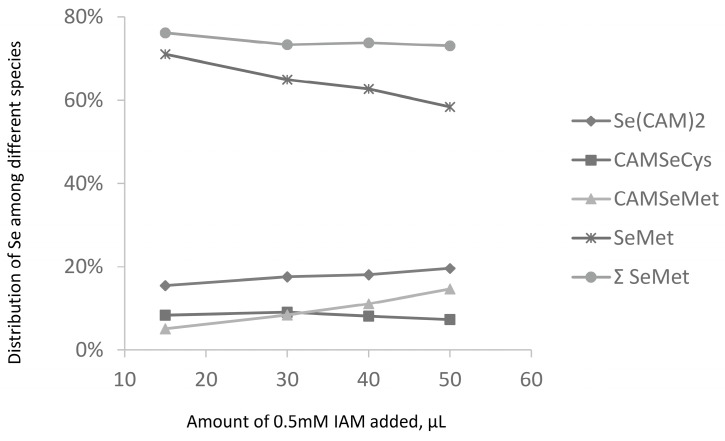
Influence of the derivatization conditions on relative intensity of proteolytic digestion products.

This species was first detected in eggs [24] and then in milk and milk-related food products [22]. The presence of inorganic selenium in selenized yeast is a sensitive topic in view of the requirement for “organic character” of selenized yeast commercial products (threshold of 1–2%) [35]. The hypotheses concerning the origin of this species include:

(i) The occurrence of selenite-binding proteins: their presence in many organisms has been well documented and a binding site consisting of two neighboring Cys residues was identified [36]; such easily-accessible sites are present in many yeast proteins and are able to scavenge (bind) inorganic selenium present in the growth medium rich in selenium;
(ii) The presence of S-Se-S bridges similar to those present in several already identified metabolites [7,37] which when reduced would expose non-SeCys selenium to the derivatization reagent. However, no CAM–Se–S-proteins could be seen in proteomics studies;
(iii) The traces of elemental (e.g., nanoparticulate) selenium or residual post-processing Se(IV) present (trapped) in water insoluble fraction. We observed that the carbamidomethylated selenium compound (Se(CAM)_2_) was readily formed from any inorganic Se, including selenate, selenide, or Se^0^ nanoparticles; and
(iv) loss of Se during derivatization of SeCys residues. This hypothesis was investigated by an increase in the iodoacetamide concentration (Figure 5). It would be expected that harsher derivatization conditions would increase the intensity of the Se(CAM)_2_ peak. However, hardly any effect of the SeCys signal was observed. No significant increase in the intensity of the Se(CAM)_2_ was observed either which would suggest that the source selenium had already been present in the sample. Note that an increase in the IAM concentration results in a decrease of selenomethionine concentration and formation of derivatization byproducts, which indicates the need for a careful optimization of the derivatization conditions should SeMet be determined together with SeCys.

### 2.6. Quantification of SeCys and Validation Strategies

The completeness of proteolytic digestion was verified by SEC-ICP MS of the digest where no residual high-molecular fraction could be observed. Therefore, selenocysteine can be quantified by the method of standard additions using CAM–SeCys standard. The recovery data at three levels are presented in Table 2. The limits of detection were 19 ng of SeCys (as Se) per gram of dry sample and the typical relative standard deviation of 6–7%. The slope of the calibration curve is similar to that obtained by standard additions of selenomethionine and quantification of the latter. These slopes are also identical to that of a calibration curve established by analyzing different concentrations of SeMet in water. These results allow the conclusion that the isocratic elution allows the use of selenomethionine standard to quantify any Se-containing compound eluting within the period of the isocratic elution.

Data acquired for the analysis of Se-rich yeast samples from different manufacturers are shown in Table 3. The sum of all the compounds accounts for the totality of selenium which can be considered as an additional element of the validation of the procedure. Note that the results obtained for sample B for the simultaneous determination of selenomethionine by this method are similar to those obtained by anion-exchange HPLC-ICP MS without derivatization; they are lower for samples A and C, thus suggesting the presence of some non-proteinaceous (water-soluble) SeMet in these samples (Table 1).

## 3. Experimental

### 3.1. Samples

Three commercial samples of selenized yeast (A, B, C) from different manufactures were used for the method development. A certified reference material, SELM-1 (National Research Council of Canada), was also analyzed to validate the total Se and selenomethionine determinations.

### 3.2. Reagents

Analytical reagent grade chemicals and LC-MS grade solvents were purchased from Sigma-Aldrich (Saint Quentin Fallavier, France) unless stated otherwise. Ultra-pure water (18 MΩ cm) obtained with a MiliQ system (Millipore, Bedford, MA, USA) was used throughout all experiments unless stated otherwise. Hydrogen peroxide from Fisher Scientific (Hampton, NH, USA) and nitric acid (INSTRA-ANALYZED) from J.T. Baker (Central Valley, PA, USA) were used for sample digestion. Protease XIV used was *Streptomyces griseus*. A standard solution of 1000 mg/L selenium was purchased from Plasma CAL standards (Teddington, UK).

### 3.3. Standards

Selenomethionine was purchased from Sigma-Aldrich. Carbamidomethylated selenocysteine (CAM-SeCys) was obtained as described elsewhere [10]. Briefly, 1.5128 mg of Se in form of SeCys_2_, powder was dissolved in 2 mL of 50 mM Tris buffer (pH 8.6). The reaction system was closed tightly and heated up to 40 °C for 15 min. Then, 50 mg of DTT (25 mg/mL) and 112 mg of iodoacetamide (66 mg/mL) in the Tris buffer were added. The mixture was left reacting for one hour. Then, 90 mg of DTT were added in 2 mL of Tris and stirred for 15 min. pH of final solution was adjusted by addition of 25 µL of HCl. Carbamidomethylated Se (Se(CAM)_2_) was synthesized similarly (SeO_2_ was suspended in the initial solution as the substrate instead of SeCys_2_).

### 3.4. Instrumentation

Digi-Prep system from SCP Science (Ottawa, QC, Canada) was used for the digestion of the samples. 5804R and 5415R centrifuges from Eppendorf (Hamburg, Germany) were used depending on the sample volume. A Branson 2510 ultrasonic bath from Emerson (Danbury, CT, USA), and an OLS 200 thermostated shaking water bath from Grant (Cambridge, UK) were used for sample incubation. pH was adjusted using a pH 213 pH meter from Hanna Instruments (Woonsocket, RI, USA). The HPLC system used was an Agilent 1200 HPLC from Agilent Technologies (Tokyo, Japan); the chromatographic columns used are listed in Table 4. The ICP mass spectrometer used was an Agilent 7700x from Agilent Technologies (Tokyo, Japan). An ESI Orbitrap LTQ Velos from Thermo Fisher Scientific (Waltham, MA, USA) was used for MS and MS/MS analysis. Both mass spectrometers were coupled with an Agilent 1200 HPLC system in order to ensure exactly the same elution conditions.

### 3.5. Procedures

#### 3.5.1. Total Selenium Determination

Ca. 0.2 g of sample was accurately weighed in a DigiPrep tube and left overnight with 2.5 mL of HNO_3_. Then, 1 mL of H_2_O_2_ was added and the sample was digested in a DigiPrep (the digestion program: 0–30 min up to 65 °C, 30–240 min-kept at 65 °C). The digests were diluted to reach the HNO_3_ concentration of 4% and analyzed by ICP MS using the optimized conditions. The quantification was carried out by the method of standard additions at four levels. The samples were analyzed in triplicate. The analytical blanks and SELM-1 were analyzed in parallel.

#### 3.5.2. Determination of Total Selenomethionine (SeMet)

A 0.2 g amount of sample was incubated with 5 mL of a protease XIV solution (20 mg protease in 30 mM Tris buffer, pH 7.5). Three consecutive incubations (17 h at temperature 37 °C) with fresh portions of the enzyme solution were carried out. After each incubation, the sample was centrifuged (10 min at 4000 rpm) and the supernatant was transferred to a separate vial to which 5 μL of β-mercaptoethanol was added. Upon the completion of the whole series, the three supernatants were pooled together and analyzed by anion exchange HPLC-ICP MS in gradient elution mode (Table 5). The quantification was carried out by the method of standard additions with SeMet at four levels. The samples were analyzed in triplicate. The analytical blanks were included in the measurements. SELM-1 was analyzed in parallel.

#### 3.5.3. Determination of Proteinaceous Selenocysteine (SeCys), Inorganic Se and SeMet

A 0.2 g amount of sample was leached three times with a fresh portion of 5 mL of water (by sonication for 1 h and centrifugation at 4000 rpm); the solution after each washing was discarded. A total of 2 mL of 0.1 M Tris buffer (pH 7.5) was added to the residue followed by addition of 30 µL of dithiothreitol (DTT) solution (0.2 M solution in 0.1 M Tris buffer, pH 7.5) and 50 µL of iodoacetamide (IAM, 0.5 M solution in Tris buffer). Then, a fresh 150 μL aliquot of the DTT solution was added and the mixture was shaken for 1 h in order to destroy excess IAM. Subsequently, 10 mL of Tris buffer and an aliquot of 1 mL of a protease solution (30 mg protease XIV in 2 mL of 100 mM Tris buffer, pH 7.5) was added to the sample. After 2 h incubation a second aliquote of 1 mL protease solution was added and the sample was incubated overnight at 37 °C. After centrifugation the supernatant was removed, filtrated on a 2 kDa filter, and analyzed by HPLC-ICP MS using chromatographic conditions given in Table 2. The quantitation of SeCys, I, organic selenium, and SeMet was carried out using the method of standard (CAM-SeCys Se(CAM)_2_ and SeMet, respectively) additions at three levels. The samples were analyzed in duplicate. The analytical blanks were measured in parallel.

## 4. Conclusions

The developed procedure allowed the quantitative recovery of the proteinaceous selenocysteine from Se-rich yeast and its separation from other species. The well-defined peak of CAM-SeCys observed by ion-paring reversed phase HPLC-ICP MS can be the basis for the quantification of this selenoamino acid. The method allows the quantification of the residual inorganic selenium which is most likely attached covalently to proteins or present in the reduced Se (0) form. Finally, SeMet can be determined on the basis of the same chromatogram and the results are in agreement with the reference method. The identification of all the peaks occurring in the chromatogram and the mass balance allows the internal validation of the procedure. However, the certification of the SeCys concentration in SELM-1 (or issuing another reference material) is necessary for the quality assurance of the analyses for SeCys in yeast.

## Figures and Tables

**Table 1:** The results of the total Se, SeMet, and water-soluble selenium species determination in the studied samples.

Sample	Total Se, ppm	Total SeMet *, ppm as Se	Water Soluble Se Species, ppm
SELM-1	1928 ± 59 **	1362 ± 49 ppm ***	276 ± 8
A	1919 ± 51	1670 ± 132	185 ± 2
B	2087 ± 70	1283 ± 52	276 ± 8
C	2292 ± 30	1676 ± 24	354 ± 5

* According to the standard AE HPLC-ICP MS method, ** certified 2059 ± 64 ppm, *** certified 1365 ± 70 ppm.

**Table 2:** Recoveries of selenocysteine (as selenium) added.

	Peak Area, ^76^Se	
Added	Found, ng/mL	Error, %
1	1.02	2.25
2.5	2.43	−7.00
5	4.98	−2.25
10	9.99	−1.00

**Table 3:** Quantification of selenium species in studied yeast samples.

Sample	Total Se, ppm	SeCys, ppm as Se	SeMet *, ppm as Se	Se(CAM)_2_, ppm as Se	Water Soluble Se, ppm	Sum of Species **	Yield, %
SELM	1928 ± 59	81 ± 11	1367 ± 93	139 ± 13	276 ± 8	1883	97.7
A	1919 ± 51	109 ± 14	1563 ± 22	97 ± 11	185 ± 2	1954	102.8
B	2087 ± 70	58 ± 7	1254 ± 70	352 ± 24	276 ± 8	1940	93.0
C	2292 ± 30	105 ± 15	1476 ± 32	203 ± 16	354 ± 5	2138	93.3

* Sum of all the SeMet forms detected by RP C8 HPLC-ICP MS; ** sum of SeCys, SeMet Se(CAM)_2_ and water soluble Se.

**Table 4:** Chromatographic columns used.

Separation Mechanism	Name	Dimensions (d × l × Particle Size)	Supplier (Location)
Size-exclusion	Superdex-75	10 × 300 mm × 13 µm	GE Healthcare (Little Chalfont, UK)
Reversed-phase C8	Alltima C8	4.6 × 250 mm × 5 µm	HiChrom (Theale, Reading Berkshire, UK)
Mixed	Hypercarb	2.1 × 100 mm × 3 µm	Thermo Scientific (Waltham, MA, USA)
Reversed-phase C18	CS Evolution AQ	2.1 × 100 mm × 2.6 µm	Intershim (Montluçon, France)
Reversed-phase C18	Zorbax Eclipse Plus	4.6 × 100 mm × 3.5 µm	Agilent (Apple Valley, MN, USA)
Anion exchange	PRPX-100	4.1 × 250 mm × 5 µm	Hamilton Robotics (Reno, NV, USA)
HILIC	Phenomenex	2.1 × 150 mm × 2.6 µm	Kinetex (Torrance, CA, USA)

**Table 5:** Chromatographic conditions used.

Separation Mechanisms (Column)	Eluent	Elution Mode	Flow Rate, mL/min	Sample Volume, µL
**Determination of selenocysteine, selenomethionine and protein-bound selenium**				
RP (C8)	A: 0.1% HFBA * in water B: 0.1% HFBA in MeOH	Gradient: 0–15 min 3% B 15–18 min 3–40% B 18–21 min 40% B 21–23 min 40–3 % B 23–30 min 3% B	0.9	10
Mixed (Hypercarb)	A: 20 mM NFPA ** in water B: ACN	Gradient: 0–10 min 0–15 % B 10–20 min 15–26% B 20–30 min 26–50% B 30–33 min 50% B 33–34 min 50–0% B 34–40 min 0% B	0.2	5
RP (C18 CS)	A: 0.1% TFA in H_2_O B: ACN	Gradient: 0–1 min 1% B 1–5 min 1–40% B 5–6.5 min 40–80% B 6.5–8 min 80% B 8–8.5 min 80–1% B 8.5–10 min 1% B	0.3	7
**Determination of SeMet**				
Anion-exchange (PRPX-100)	A: 20 mM CH_3_COOH- 10 mM triethylamine (pH 4.7) B: 200 mM CH_3_COOH- 100 mM triethylamine (pH 4.7)	0–5 min: 0% B 5–30 min: 0–100% B 30–40 min: 100–0% B	0.7	20
**Identification of selenium species by ESI MS**				
SEC (Superdex-75)	100 mM ammonium acetate, pH 7.5	isocratic	0.9	100
HILIC (Phenomenex)	A: 90:10 ACN: 50 mM ammonium acetate B: 40:50:10 ACN/H_2_O/50 mM ammonium acetate	Gradient: 0–2.5 min 0% B 2.5–10 min 0–100% B 10–12.5 min 100% B 12.5–12.6 min 100–0% B 12.6–25 min 0% B	0.5	10
RP (C18 Zorbax)	0.1% FA in 5% methanol	isocratic	1	10

* Heptafluorobutyric acid; ** nonafluoropentationic acid.
